# On the different methods of micrographic surgery and their differences in the visualization of the tumor and surgical margin, and in the contribution to clinical and oncological aspects^☆^^☆☆^

**DOI:** 10.1016/j.abd.2020.03.003

**Published:** 2020-05-11

**Authors:** Sandro Simão Corrêa Filho

**Affiliations:** Private Clinic, Blumenau, SC, Brazil

Dear Editor,

Micrographic surgery was developed in the 1930s by Dr. Friedrich Mohs, using the *in vivo* tissue fixation method. In 1970, Stegman and Tromovitch published a series of cases using *ex vivo* fixation. In 1995, the Munich method was described.

Since then, surgeons have been constantly learning these techniques.^1^, ^2^, ^3^, ^4^

In the study by Portela et al.^5^ a new form of debulking assessment was described, but it is identical to the Munich method previously described in the literature. The concepts of margin and surgical border are imprecise. The authors illustrate an essential feature of the Munich method: the possibility of assessing the tumor–surgical margin relationship and observation of the tumor. Thus, it is possible to better demonstrate the subtype, cytological aspects, and tumor architecture, which have clinical and oncological relevance and are important for decision making. These factors gain importance in tumors with rarer histology and with greater metastatic potential; it also facilitates the identification of perineural invasion. In turn, peripheral methods evaluate only the surgical border, and do not observe the tumoral core. Although bread-loafing of the paraffin block is performed during debulking, the sample is smaller and the results are not available in the trans-operative period, given the time required for paraffin embedding and processing. A drawback of the fresh method is the greater chance of technical artifacts (Table 1).

**Table 1:** Comparison between some characteristics of the Munich method and peripheral methods^a^

	Munich method	Peripheral methods (Mohs, Tübingen, muffin)
Observation of the tumor core	Yes	No
Tumor analysis	Yes	No (only if there is tumor involvement of the surgical border)
Evaluation of the cutaneous tumor site^b^	Yes	No
Observation of the tumor-surgical margin relationship	Yes	No
Analysis of tumor cytology (*e.g*., mitotic figures)	Yes	No (only if there is tumor involvement of the surgical border)
Assessment of perineural involvement	Easier	More difficult
Number of glass slides	Greater	Smaller

a Even if a previous biopsy of the affected area is performed, there may be a discrepancy between the data from the incisional biopsy and the posterior excision due to sampling, as pointed out by Portela et al.^5^

b Important in ill-defined tumors or scars.

The author of this correspondence highlights the importance of broadening the discussion of the technical and laboratory details of the various forms of micrographic surgery, including the implications of each technique for the clinical and oncological data.

## Financial support

None declared.

## Author's contributions

Sandro Simão Corrêa Filho: Approval of the final version of the manuscript; conception and planning of the study; drafting and editing of the manuscript; critical review of the literature; critical review of the manuscript.

## Conflicts of interest

None declared.
